# Milk Thistle Oil Extracted by Enzyme-Mediated Assisted Solvent Extraction Compared with n-Hexane and Cold-Pressed Extraction

**DOI:** 10.3390/molecules28062591

**Published:** 2023-03-13

**Authors:** Longlong Liu, Hua Zhang

**Affiliations:** Agronomy of Food Science and Technology, Yanbian University, Yanji 133002, China

**Keywords:** milk thistle oil, silymarin, response surface methodology, antioxidant properties

## Abstract

Silymarin and milk thistle oil have unique biological benefits; however, applying silymarin to milk thistle oil remains a challenge. In this research, the content of silymarin in milk thistle oil conditions using enzyme-mediated solvent extraction was investigated and optimized by response surface methodology. The optimal extraction conditions using enzyme-mediated solvent extraction were as follows: the enzyme-added content was 3.06 mg/mL, the enzymatic hydrolysis temperature was 55.09 °C, and the enzymatic hydrolysis time was 66.28 min. Oil extracted by the enzyme-mediated assisted solvent was further compared with those extracted with n-hexane and cold pressing. Results indicated that the oil extraction using the enzyme-mediated assisted solvent had a lower acid value (2.20 ± 0.01 mg/g) and the highest α-tocopherol content (0.62 ± 0.00 mg/g), total phenols (7.67 ± 0.01 mg/g), and flavonoids (1.06 ± 0.13 mg/g). Furthermore, the antioxidant capacity of milk thistle oils was further investigated. The results showed that the enzyme-mediated assisted solvent-extracted oil had the strongest antioxidant capacity with lower lipid oxide content. Therefore, enzyme-mediated solvent extraction is an excellent method for extracting milk thistle oil.

## 1. Introduction

Milk thistle (*Silybum marianum* L. Gaertn.) is a critical medicinal plant from the family Asteraceae [1,2]. Milk thistle has been widely used in the food industry as an important raw material for cooking oil in recent years in the world [3]. It enriches silymarin [4,5,6,7,8,9], lipids (about 25~30%) [10,11,12], proteins [13], and polysaccharides [14]. Silymarin not only has excellent performance in the treatment of the liver, but has physiological functions such as antioxidant functions [15,16], anti-inflammatory functions [17,18,19], anti-cancer functions, etc. [20,21]. Hence, silymarin is widely used in the pharmaceutical industry. Milk thistle oil is rich in unsaturated fatty acids (56% polyunsaturated and 21% monounsaturated) [22], vitamin E [5], flavonoids, and phenolics, so it can be used in anticholesterol diets for cardiovascular disease prevention [23].

Milk thistle oil is generally extracted by single chemical solvent [9], microwave extraction [24,25], supercritical fluid [26] and other methods. Solvent extraction often requires relatively large quantities of solvents, and post-processing is more complicated. Improper handling can easily lead to the loss of nutrients and even the introduction of toxins [27,28]. Supercritical and microwave-assisted extractions have been recently applied to lipid extraction; nevertheless, these methods have not yet been widely utilized in the process of practical production due to higher operation cost (industrial level) and difficult operation (technical level).

Enzyme-mediated assisted solvent extraction is also widely used for lipid extraction. Compared with traditional extraction methods, this method has a high extraction efficiency, high content of physiologically active substances, and greatly enhanced antioxidant capacity [29,30,31,32].

Although various research on milk thistle oil, silymarin, and enzyme-mediated solvent has been published, there is no report on the utilization of the enzymatic approach to increase the silymarin content of milk thistle oil. This study has sought to investigate the effect of extraction on the content of silymarin in milk thistle oil by the enzyme-mediated solvent extraction method. The extraction conditions were therefore optimized, and the properties of the oils extracted by different methods were evaluated and compared. Furthermore, physical and chemical properties, antioxidant capacity, and active substances were also evaluated and compared.

## 2. Results and Discussion

### 2.1. Optimization of the Conditions for the Enzyme-Mediated Solvent Extraction

The enzyme-mediated assisted solvent extraction method has more advantages than other methods based on nutrient retention, which is more suitable for nutrient-oriented oil extract such as that from milk thistle oil. Different factors such as enzyme added content, enzymatic hydrolysis temperature, and enzymatic hydrolysis time that influenced the content of silymarin in milk thistle oil were compared.

**Figure 1:**
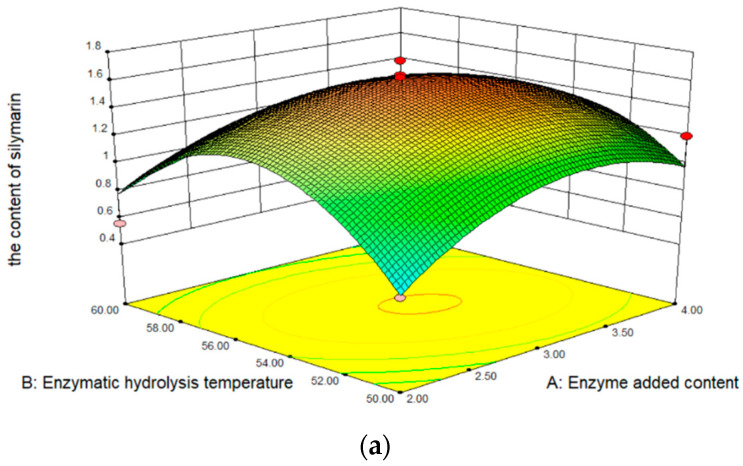

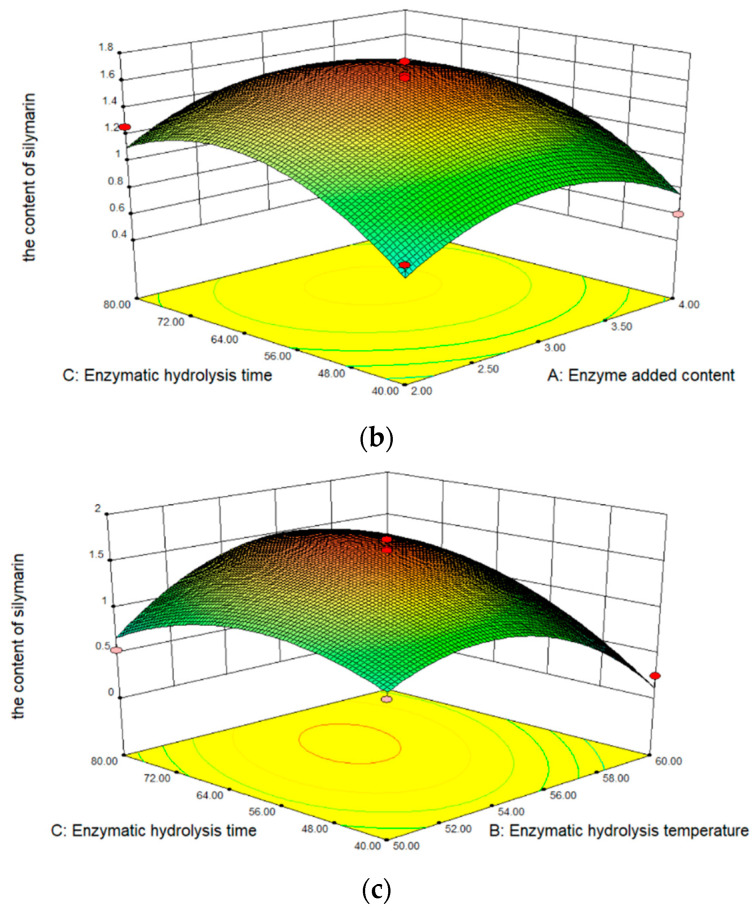
Three-dimensional response surface for the content of silymarin. (**a**) Interaction of temperature and content; (**b**) interaction of time and content; (**c**) interaction of time and temperature.

The results obtained from the RSM experiments are summarized in Table 1. The content of silymarin in oil is 0.250 to 1.739 mg/100 mg, depending on the extraction conditions. In analyzing the significance of coefficients for the content of silymarin, the content change substantially linearly enzyme added content, enzymatic hydrolysis temperature, and enzymatic hydrolysis time. In addition, the effect of enzymatic hydrolysis temperature on the content of silymarin was the most significant extraction factor. Figure 1a shows the effect of enzymatic hydrolysis temperature and enzyme added content on the content of silymarin extraction efficiency. The content of silymarin increased with the increase of the temperature and enzyme content, but the content of silymarin begins to decrease when the temperature exceeds 55 °C, due to the inactivation of cellulase. The content of silymarin increased with the increase in time (Figure 1b). Furthermore, as the enzyme added content increased, the content of silymarin did not increase continuously, but first increased and then decreased (Figure 1c). The reason for this phenomenon is that after too much cellulase is added, the silymarin seed powder is closely combined with the enzyme, and part of the surface is covered, preventing the dissolution of silymarin. 

ANOVA was used to examine the adequacy of the suggested models, including the effects of the linear, quadratic, or interaction coefficients of the content of silymarin, and the results are presented in Table 2. Further analysis showed that the response and processing variables are fitted to each other in a quadratic regression equation. Y = 1.61 + 0.039A − 0.064B + 0.22C − 0.15AB + 6.00E − 003AC + 0.30BC − 0.32A^2^ − 0.56B^2^ − 0.35C^2^. Y is the content of silymarin in the milk thistle oil. The significance of the model (*p* < 0.01) showed that the regression model was statistically relevant to enzyme added content, enzymatic hydrolysis temperature, and enzymatic hydrolysis time. Moreover, the lack of fit was not significant (*p* > 0.05), which indicated that the model of the primary objectives should fit the experimental data. The determination correlation coefficient (Adj-R^2^) was 0.86, indicating that 86% of the variability in the response could be explained by the model.

Thus, using RSM with interactive calculations in the selected range, optimal extraction conditions were as follows: the enzyme added content was 3.06 mg/mL, the enzymatic hydrolysis temperature was 55.09 °C, and the enzymatic hydrolysis time was 66.28 min. The theoretical maximum content of silymarin was 1.6467 mg/100 mg of oil under optimal conditions. To verify the reliability of this method, the optimal extraction conditions were applied for milk thistle oil extraction. Three parallel experiments verified that the silymarin content was 1.6322 ± 0.0301 mg/100 mg, which was close to the predicted value, and this result would indicate that the models were well fitted.

### 2.2. Comparison of Total Phenolic, Flavonoids, and α-Tocopherol Content of Milk Thistle Oil

**Figure 2:**
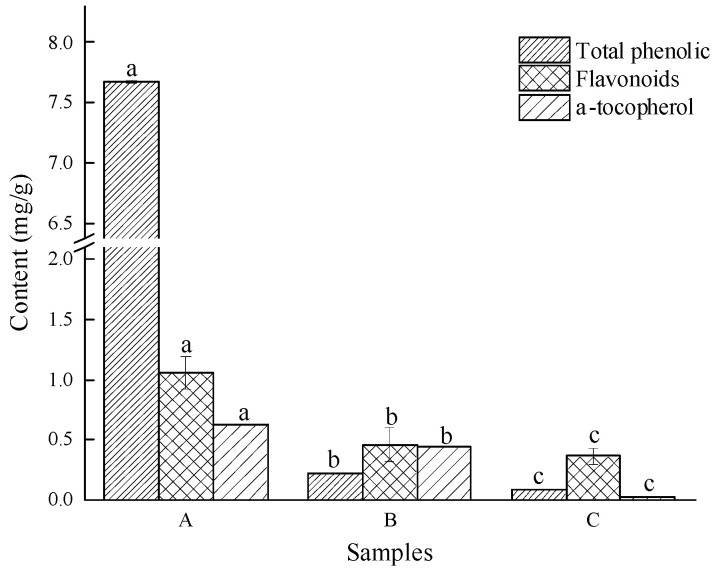
Content of total phenols, flavonoids, and α-tocopherol. Note: A is enzyme-mediated solvent extraction, B is n-hexane extraction, and C is cold-pressed extraction. Values in the different samples with different letters are significantly different (*p* < 0.05).

Phenols, flavonoids, and α-tocopherol are important active ingredients in edible oils that play an antioxidant role. Generally, the higher the content of these substances in edible oils, the stronger the antioxidant capacity and the higher the oxidative stability of oils. In this research, the content of active substances in milk thistle oil extracted by enzyme-mediated assisted solvent was significantly higher than that of the other two groups (*p* < 0.05) (Figure 2), which was closely related to its unique extraction process. The content of α-tocopherols in cold-pressed milk thistle oil was extremely low—only 0.15 mg/g—indicating that the refining process of edible oil would lead to serious loss of natural antioxidants.

### 2.3. Comparison of Antioxidant Activity of Milk Thistle Oil

**Figure 3:**
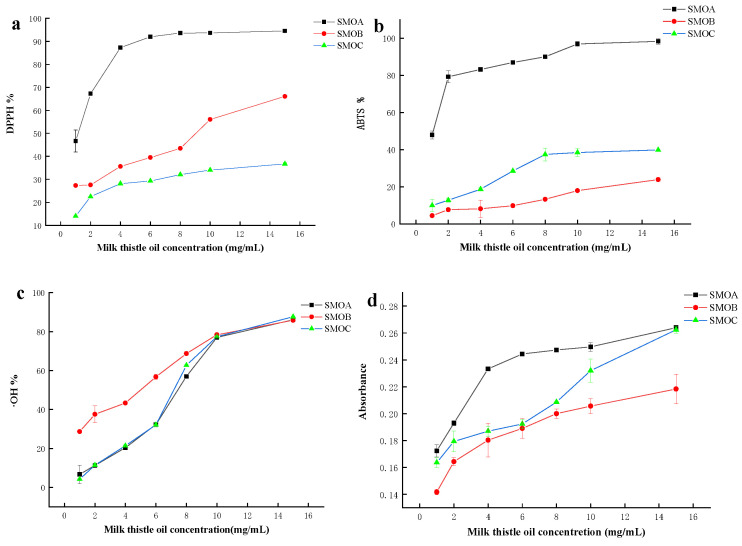
Antioxidant activity of milk thistle oils. (**a**) DPPH radical scavenging, (**b**) ABTS radical scavenging, (**c**) hydroxyl radical scavenging ability, and (**d**) total antioxidant capacity.

Antioxidant activity of milk thistle oil was assessed by four valid methods, i.e., DPPH, ABTS, ∙OH scavenging rate, and total antioxidant capacity. As shown in Figure 3, the extraction method has a significant effect on the antioxidant activity of milk thistle seed oil. The results of DPPH, ABTS, and total antioxidant capacity showed that the antioxidant capacity of enzyme-mediated solvent extraction was significantly higher than the other two groups (*p* < 0.05) (Figure 3a,b,d). Similar results were reported for walnut oil [33], oat bran oil [34], and grape seed oil [35]. Generally, the high antioxidant activity of edible oil could be attributed to its affluent endogenous antioxidant ingredients such as tocopherol, polyphenol, flavonoid, etc., which can scavenge the generation of free radical and active oxygen [36]. In this work, antioxidant activity shows a correlation with the total phenolic content, total flavonoids, and tocopherol content. However, there is an insignificant correlation between ∙OH scavenging rate and the above active substances (Figure 3c).

### 2.4. Fatty Acid Composition of Milk Thistle Oil

The fatty acid composition is a crucial identity characteristic of edible oil, which has a great influence on its physicochemical and nutritional properties. The fatty acid composition of milk thistle seed oils extracted by different methods is shown in Table 3. In all oil samples studied, 12 types of fatty acids were identified according to GC analysis. Linoleic (C18:2n6c, 50.51–64.22%), oleic (C18:1n9c, 16.80–22.72%), palmitoleic acid (C16:1n7, 0.06–0.09%), arachidic acid (C20:1, 0.96–1.04%), and linolenic acid (C18:3n3, 0.22–0.38%) were the most predominant unsaturated fatty acids, which account for more than 80% of the total fatty acids. Except for cold pressing, the extraction method had no significant effect on the fatty acid composition. This phenomenon is due to the deacidification, deodorization, and high-temperature treatment of cold-pressed oil, which is not conducive to the retention of unsaturated fatty acids [37].

### 2.5. Comparison of POV, TBARS, AV, and P-AnV of Milk Thistle Oil

AV, POV, TBARS, and *P*-AnV were compared to monitor lipid oxidation status. As shown in Table 4, the AV of the oil extracted by the enzyme-mediated solvent is slightly lower than that of the oil extracted by n-hexane and higher than that of the oil extracted by cold pressing. The POV of the oil extracted by enzyme-mediated assisted solvent was the lowest. The TBARS and *p*-AnV of oil extracted by enzyme-mediated assisted solvent were significantly higher than those extracted by n-hexane and cold press (*p* < 0.05). These results suggested that enzyme-mediated solvent extraction of milk thistle oil contributes to better lipid quality.

## 3. Materials Procedures

### 3.1. Material and Reagents

Milk thistle seed was purchased from Hongshuo Biotechnology Co., Ltd. (Anhui, China). Milk thistle oil (cold pressed oil; raw material and milk thistle seeds from the same source) is provided by Yanbian Yuanshan Oil Technology Co., Ltd. (Jilin, China). α-tocopherol standard, standards of fatty acid methyl esters (FAME), and malonaldehyde bis were purchased from Sigma-Aldrich Chemical Co. Ltd. (Shanghai, China). Hexane, methanol, and ethanol are HPLC grade and were obtained from Aladdin Biochemical Technology Co., Ltd. (Shanghai, China). Other solvents and reagents were of analytical grade and from Sinopharm Chemical Reagent Co., Ltd. (Shanghai, China).

### 3.2. Instruments and Equipment

Equipment used included a high speed centrifuge (Allegra 64R, Beckman Coulter, Inc., California, CA, USA), an electronic balance (ML-T, METTLER TOLEDO, Inc., Zurich, Switzerland), a rotary evaporator (Rotavapor^®^ R-100, BÜCHI Labortechnik AG, Flawil, Switzerland), an ultraviolet spectrophotometer (Evolution 220, Thermo Fisher Scientific, Inc., Waltham, MA, USA), a thermostatic water bath (HH-M4, HerryTech Co., Ltd., Shanghai, China), gas chromatography (Track 1300, Thermo Fisher Scientific, Inc., Waltham, MA, USA), and a high-performance liquid chromatography (LC-2010A, Shimadzu (China) Co., Ltd., Shanghai, China).

## 4. Methods 

### 4.1. Extraction by Enzyme-Mediated Solvent

In a 1000 mL conical flask, weigh 100 g of milk thistle seed powder and mix with 300 mL of solution (n-hexane: ethanol, 2:1, *v*/*v*). The added cellulase measuring 3.06 mg/mL reacts under appropriate conditions, and the samples were extracted by shaking at 25 °C for 2 h. After filtering twice, the mixture was rotary evaporated at 45 °C until no solvent flowed out, and the oil was then flushed with nitrogen to eliminate the residue hexane. Based on single factor experiments, response surface methodology (RSM) was evaluated with three independent variables: enzyme-added content (A), enzymatic hydrolysis temperature (B), and enzymatic hydrolysis time (C) (Table 5). The content of silymarin (Y) was chosen as a response, and 17 experiments (Table 1) were performed in a randomized order. The three-level Box–Behnken rotatable design was designed by Design-Expert^®^ 8.0.6 software (Stat-Ease inc., Minneapolis, MN, USA) for modeling and optimization of the effects of the process factors on the content of silymarin. The determination of silymarin content refers to the method in Section 4.3.

### 4.2. Extraction with n-Hexane

100 g of milk thistle seed powder was mixed with 1200 mL of n-hexane in a beaker. The mixture was agitated at 25 °C for 2 h using a mechanical stirrer and then centrifuged at 1860× *g* for 15 min. The supernatant was filtered under a vacuum. The filtered liquid was collected and evaporated at 45 °C in a rotary evaporator to remove n-hexane. The oil was flushed with nitrogen to remove the residue hexane [9].

### 4.3. The Content of Silymarin

Silymarin content was determined using HPLC. Briefly, 100 mg of milk thistle oil is taken and adsorbed for 2 min on an activated C18 SPE column (1000 mg, 6 mL), after which 10 mL of n-hexane is added for elution. The receiving solution is then discarded, and the process is repeated with 10 mL of ethanol. At 45 °C, the ethanol receiver liquid is spun dry in an evaporator. It is then dissolved in a 2 mL solution of methanol and detected using HPLC. The chromatographic detection conditions are as follows [38]: chromatographic column—Agilent TC-C18(2), 250 × 4.6 mm, 5 μm; mobile phase A—methanol (chromatographic grade) and mobile phase B—methanol (20% ultrapure water); injection volume 20 μL; column temperature 45 °C; flow rate, 0.6 mL/min; and detection wavelength, 288 nm. The elution gradient was 0–8 min, 80% B; 8–10 min, 10% B. In addition, silibinin was used as the standard to draw a standard curve, and the regression equation was Y = 7.33472 × 10^7^ X − 514722, R^2^ = 0.9998.

### 4.4. Fatty Acids Composition

The oils used for the determination of fatty acid composition were methylated following the BF_3_-methanol method as described in the standard of ISO:5509 (2000). The type of each fatty acid was finally determined by comparison with the retention time of the fatty acid methyl ester standard. The gas chromatograph conditions were as follows: capillary column—polycyanopropyl siloxane strong polar stationary phase (100 m × 0.25 mm, 0.2 μm); detector—gas chromatography-flame ionization detection (GC-FID) detector; injector temperature, 250 °C; detector temperature, 260 °C; temperature programming, 70 °C at 10 °C/min to 160 °C, for 10 min, then 2.5 °C/min, warm to 225 °C for 30 min; carrier gas—nitrogen split ratio of 100:1; and injection volume—1 μL.

### 4.5. Determination of Total Phenolic Content

The total phenolic content was determined following the method of Yoo et al. [39]. Briefly, 1 mL of oil sample was combined with 1 mL of Folin-Ciocalteu reagent, and 10 mL of 7.5% Na_2_CO_3_ solution was added after 5 min. Use deionized water to make a final volume of 25 mL of the reaction mixture; incubation was carried out for 2 h at room temperature, and absorbance values were recorded at 765 nm. Total phenolic contents were then reported as mg gallic acid equivalent (GAE)/100 g after comparing the sample results with those of known concentrations of gallic acid solutions.

### 4.6. Determination of Flavonoids Content

Weigh 0.4000 g milk thistle oil into a centrifuge tube, add 70% ethanol to 2 mL, mix well and dissolve, extract in a water bath at 50 °C for 30 min, centrifuge at 4000 r/min for 10 min, take the supernatant, and repeat the extraction three times. Combine the three supernatant solutions, dilute the volume to 4 mL, add 2 mL of 0.1 mol/L AlCl_3_ and 1 mL of NaAc-HAc buffer solution with pH 5.2, develop color in a water bath at 40 °C for 10 min, and measure the absorbance at 400 nm. All experiments were measured in pa rallel three times. The flavonoid contents were then reported as tannins equivalent/100 g after comparing the sample results with those of known concentrations of tannins solutions [40].

### 4.7. Determination of α-Tocopherol

The content of α-tocopherol was determined by normal-phase high-performance liquid chromatography. Briefly, weigh 1.0 g of the sample, add 0.1 g of BHT, dissolve it with n-hexane and dilute it to 25 mL, pass through a 0.45 μm filter membrane, and use the HPLC method for the determination.

HPLC measurement conditions were as follows: Agilent 2010-A chromatograph equipped with UVD detector; chromatographic column—Kromat Universil SiO_2_ (4.6 × 250 mm, 5 μm); column temperature, 30 °C; detection wavelength, 292 nm; flow rate, 0.8 mL/min; and injection volume, 20 μL. Calculation of α-tocopherol content in samples was performed using α-tocopherol standards.

### 4.8. Antioxidant Activity Assays of Milk Thistle Seed Oil

#### 4.8.1. DPPH Radical Scavenging Assay

Oil extract measuring 200 μL was diluted with 1.8 mL of methanol, and then 2 mL of 0.1 mmol/L DPPH methanolic solution was added. The mixture was vortexed for 2 min and stored in the dark for 2 h. The absorbance of the mixture was recorded at 517 nm by a UV–Vis spectrophotometer against a blank solution without DPPH. The DPPH antioxidant activity was expressed as percentage inhibition, following the formula in the literature [41]:

$$\;\mathrm{DPPH}\;\%=\frac{\;A_{\mathrm{blank}\;}\;-A_{\mathrm{sample}\;}}{A_{\mathrm{blank}\;}}\times 100$$

where A is the absorbance at 517 nm.

#### 4.8.2. ABTS Radical Scavenging Assay

Briefly, equal volumes of potassium persulphate solution (2.6 mM) and 2.2-azino-bis (3-ethylbenzothiazoline-6-sulphonic acid) (ABTS) solution (7.4 mM) were mixed, and the solution was kept in the dark for 16 h [42]. ABTS measuring 3 mL was mixed with 0.3 mL of the sample solution, and, after standing at room temperature for 6 min, the absorbance at 734 nm was quickly measured as A_i_. In the same way, 3 mL of absolute ethanol (95%) was mixed with 0.3 mL of the sample solution, and the absorbance was measured as A_j_. Then, take 3 mL of ABTS and mix it with 0.3 absolute ethanol, measure the absorbance value A_0_, and calculate the ABTS radical as follows:

$$ABTS\%=\frac{A_{0}-\left(A_{i}-A_{j}\right)}{A_{0}}\times 100$$

#### 4.8.3. Hydroxyl Radical Scavenging Ability

Milk thistle oil was prepared with absolute ethanol into samples with mass concentrations of 1, 2, 4, 8, 10, and 15 mg/mL, respectively. Measure 2 mL of milk thistle oil, add 2 mL of 6 mmol/L FeSO_4_ and H_2_O_2_ in turn, mix well and let stand for 10 min, then add 2 mL of 6 mmol/L salicylic acid, mix well, and let stand for 30 min. The absorbance at 510 nm wavelength was measured as A_i_, and the absorbance of salicylic acid replaced by distilled water was recorded as A_j_. The absorbance of milk thistle oil replaced by distilled water was recorded as A_0_. Calculate the scavenging rate of hydroxyl radicals according to the following [43,44]:

$$\;\mathrm{Hydroxyl}\;\mathrm{radical}\;\%=1-\frac{A_{i}-A_{j}}{A_{0}}$$

#### 4.8.4. Total Antioxidant Capacity

Milk thistle oil was prepared with absolute ethanol to prepare samples with mass concentrations of 1, 2, 4, 8, 10, and 15 mg/mL, respectively. Take 0.4 mL milk thistle oil, and add 4 mL phosphomolybdic acid reagent (28 mmol/L sodium phosphate, 0.6 mol/L concentrated sulfuric acid, and 4 mmol/L ammonium molybdate) in a constant temperature water bath at 95 °C for 90 min. The absorbance at a wavelength of 695 nm was measured [43].

### 4.9. Peroxide Value and Thiobarbituric Acid Reactants Measurement

Peroxide value (POV) was performed according to the American Oil Chemists’ Society (AOCS) Method Cd 8b-90 [45] with slight modifications. Briefly, 2.0 g of milk thistle oils were dissolved in 6 mL of a mixed solution (acetic acid: chloroform, 3:2, *v*/*v*). Saturated potassium in 1 mL iodine solution and 100 mL of distilled water were added to the mixture and shaken. Then, the solution was titrated with 0.1 N of sodium thiosulfate until the yellow iodine color disappeared. Immediately, 1 mL of starch indicator solution was added by shaking to extract iodine from the chloroform layer and again titrated until the blue color disappeared. A blank determination was performed with the same procedure. the POV is calculated by the following:

$$POV=\frac{\left(V-V_{0}\right)\times c}{2\times m}\times 1000$$

where V is the volume of sodium thiosulfate consumed by the sample (mL), V_0_ is the volume of sodium thiosulfate consumed by the blank reagent (mL), c is the concentration of sodium thiosulfate standard solution (N), and m is sample quality (g).

The thiobarbituric acid reactants measurement (TBARS) was determined according to the method described by the AOCS Method Cd 19-90 [46], and malonaldehyde bis was used as the standard to do the standard curve to calculate the content in the samples.

### 4.10. Acid Value and P-Anisidine Value Measurement

Acid value (AV) was calculated according to the AOCS Method Cd 3d-63 [47]. Oil measuring 1.00 g was mixed with 15 mL ethanol, and it was titrated with 0.25 N of potassium hydroxide solution using phenolphthalein as a color indicator, which causes pink coloring. The AV is calculated with the following formula:

$$AV=\frac{V\times C\times 56.1}{W}$$

where V is the volume of KOH used for titration of the oil samples, C is KOH concentration, and W is the weight of the oil sample in grams.

*P*-Anisidine value (*P*-AnV) was determined according to the procedure of the European Pharmacopeia, 8th edition [48]. A sample of 0.50 g oil was dissolved in trimethylpentane and diluted to 25 mL with the same solvent. The absorbance was measured at a wavelength of 350 nm using trimethylpentane as the compensation oil. Then 1 mL of 25 g/L solution of *p*-anisidine in glacial acetic acid was added to the previously prepare oil sample solution (5 mL), shaken, and stored protected from light. The reference solution was prepared with trimethylpentane instead of the oil sample solution. After 10 min, absorbance was measured at 350 nm using the reference solution as the compensation oil.

### 4.11. Statistical Analysis

All data were analyzed in Excel for mean and variance analysis. The experimental values were expressed by MEAN ± SD. One-way analysis of variance (ANOVA) was performed using SPSS Statistics 22 software (IBM, New York, NY, USA), and Origin 2018C (OriginLab Corporation, Northampton, MA, USA) was used for data mapping. Response surface analyses were carried out using Design-Expert^®^ software 8.0.6 (Stat-Ease inc., Minneapolis, MN, USA).

## 5. Conclusions

To explore milk thistle oil extraction for its high nutrient values, this work has investigated the effect of enzyme-mediated solvent extraction. The results showed that the enzyme-mediated solvent extraction method was a suitable method for the extraction of milk thistle oil with higher silymarin content. Moreover, it gave a better lipid quality with a lower acid value, the lowest peroxide value, thiobarbituric acid reactant, and *p*-anisidine value and the highest α-tocopherol, total phenolics, and flavonoids. In addition, enzyme-mediated assisted solvent-extracted milk thistle oil had the best oxidative stability. This research provided a novel approach to the combined use of milk thistle oil and silymarin.

## Figures and Tables

**Table 1:** Response surface experimental design and results.

Number	A (mg/mL)	B (°C)	C (min)	Y (mg/100 mg Oil)
1	2	50	60	0.5873
2	4	50	60	1.2027
3	2	60	60	0.5533
4	4	60	60	0.5733
5	2	55	40	0.7760
6	4	55	40	0.6033
7	2	55	80	1.2633
8	4	55	80	1.1147
9	3	50	40	0.7667
10	3	60	40	0.2500
11	3	50	80	0.5413
12	3	60	80	1.2133
13	3	55	60	1.4460
14	3	55	60	1.7393
15	3	55	60	1.6373
16	3	55	60	1.6180
17	3	55	60	1.6193

**Table 2:** ANOVA of extraction factors on the lipid recovery rate.

Source	Sum of Squares	df	Mean Square	F Value	*p*-Value
Model	3.41	9	0.38	10.55	0.0026 **
A	0.012	1	0.012	0.34	0.5762
B	0.032	1	0.032	0.90	0.3746
C	0.38	1	0.38	10.51	0.0142
AB	0.089	1	0.089	2.47	0.1601
AC	1.440 × 10^4^	1	1.440 × 10^4^	4.013 × 10^3^	0.9513
BC	0.35	1	0.35	9.84	0.0164 *
A^2^	0.43	1	0.43	11.88	0.0107 *
B^2^	1.34	1	1.34	37.41	0.0005 **
C^2^	0.53	1	0.53	14.75	0.0064 **
Residual	0.25	7	0.036		
Lack of Fit	0.21	3	0.069	6.19	0.0553
Pure Error	0.045	4	0.011		
Cor Total	3.66	16			

Note: “*” represents significant difference (*p* < 0.05); “**” represents a significant difference (*p* < 0.01).

**Table 3:** Fatty acid composition of milk thistle oil.

Fatty Acids	Enzyme-Mediated Solvent Extraction	Hexane Extraction	Cold Press
C14:0	0.12 ± 0.01 ^a^	0.12 ± 0.02 ^a^	0.09 ± 0.01 ^b^
C16:0	8.88 ± 0.03 ^a^	8.85 ± 0.02 ^a^	7.55 ± 0.94 ^b^
C16:1n7	0.09 ± 0.00 ^a^	0.07 ± 0.01 ^b^	0.06 ± 0.01 ^b^
C17:0	0.08 ± 0.01 ^a^	0.07 ± 0.00 ^a^	0.07 ± 0.01 ^a^
C18:0	4.08 ± 0.01 ^b^	4.34 ± 0.03 ^b^	5.14 ± 0.55 ^a^
C18:1n9c	16.80 ± 0.01 ^a^	17.07 ± 0.02 ^a^	22.72 ± 2.81 ^a^
C18:2n6C	64.22 ± 0.04 ^a^	63.21 ± 0.04 ^a^	50.51 ± 6.25 ^b^
C20:0	2.13 ± 0.03 ^b^	2.32 ± 0.02 ^b^	3.32 ± 0.05 ^a^
C20:1	0.96 ± 0.02 ^a^	1.03 ± 0.02 ^a^	1.04 ± 0.13 ^a^
C18:3n3	0.36 ± 0.00 ^a^	0.38 ± 0.01 ^a^	0.22 ± 0.03 ^b^
C22:0	1.79 ± 0.03 ^b^	2.01 ± 0.02 ^a^	2.23 ± 0.27 ^a^
C24:0	0.50 ± 0.01 ^b^	0.53 ± 0.00 ^b^	0.72 ± 0.08 ^a^
SFA	17.58 ± 0.12 ^a^	18.24 ± 0.11 ^a^	19.12 ± 1.91 ^a^
MUFA	17.85 ± 0.04 ^b^	18.17 ± 0.05 ^b^	23.82 ± 2.9 ^a^
PUFA	64.58 ± 0.04 ^a^	63.59 ± 0.05 ^a^	50.72 ± 6.28 ^a^

Note: Results are the mean ± standard deviation (*n* = 3). Values in the same row with different letters are significantly different (*p* < 0.05). SFA—saturated fatty acid; MUFA—monounsaturated fatty acid; PUFA—polyunsaturated fatty acid.

**Table 4:** AV, POV, TBARS, and *P*-AnV of milk thistle oil.

Indicators	Enzyme-Mediated Assisted Solvent	Hexane Extraction	Cold Press
AV (mg/g)	2.20 ± 0.01 ^a^	2.23 ± 0.11 ^a^	1.03 ± 0.03 ^b^
POV (mmol/kg)	0.24 ± 0.00 ^b^	0.24 ± 0.00 ^b^	0.82 ± 0.10 ^a^
TBARS (mg/kg)	0.21 ± 0.02 ^b^	0.37 ± 0.03 ^a^	0.24 ± 0.01 ^b^
*p*-AnV	26.28 ± 0.43 ^c^	36.76 ± 0.28 ^b^	44.00 ± 0.32 ^a^

Note: Results are the mean ± standard deviation (*n* = 3). Values in the same row with different letters are significantly different (*p* < 0.05).

**Table 5:** Analytical factors and levels for response surface method analysis.

Factors	Coded Level		
	−1	0	1
Enzyme-added content (mg/mL)	2	3	4
Enzymatic hydrolysis temperature (°C)	50	55	60
Enzymatic hydrolysis time (min)	40	60	80
